# Hydrolytic Degradation of Comb-Like Graft Poly (Lactide-co-Trimethylene Carbonate): The Role of Comonomer Compositions and Sequences

**DOI:** 10.3390/polym11122024

**Published:** 2019-12-06

**Authors:** Xuefei Leng, Wenwen Zhang, Yiying Wang, Yanshai Wang, Xiaoqing Li, Zhiyong Wei, Yang Li

**Affiliations:** State Key Laboratory of Fine Chemicals, Department of Polymer Science and Engineering, Liaoning Key Laboratory of Polymer Science and Engineering, School of Chemical Engineering, Dalian University of Technology, Dalian 116024, China; lengxuefei@dlut.edu.cn (X.L.); smiligzw@mail.dlut.edu.cn (W.Z.); wangyiying@dlut.edu.cn (Y.W.); wangyanshai@dlut.edu.cn (Y.W.); lixiaoqing19810930@163.com (X.L.)

**Keywords:** hydrolytic degradation, graft copolymer, comonomer compositions, sequence structures, polyesters

## Abstract

The effect of sequence on copolymer properties is rarely studied, especially the degradation behavior of the biomaterials. A series of linear-comb block, gradient, random copolymers were successfully achieved using hydroxylated polybutadiene as the macroinitiator by simple ring-opening polymerization of l-lactide (l-LA) and 1,3-trimethylene carbonate (TMC). The hydrolytic degradation behaviors of the copolymers were systemically evaluated by using nuclear magnetic resonance (NMR), gel permeation chromatography (GPC), differential scanning calorimeter (DSC), and scanning electron microscopy (SEM) to illustrate the influences of comonomer compositions and sequence structures. The linear-comb block copolymers (lcP(TMC-*b*-LLA)) with different compositions had different degradation rates, which increased with l-LA content. Thermal property changes were observed with decreased *T*_m_ and increased Δ*H*_m_ in all block copolymers during the degradation. To combine different sequence structures, unique degradation behaviors were observed for the linear-comb block, gradient and random copolymers even with similar comonomer composition. The degradation rates of linear-comb PLLA-gradient-PTMC (lcP(LLA-grad-TMC)) and linear-comb PLLA-random-PTMC (lcP(LLA-ran-TMC)) were accelerated due to the loss of regularity and crystallinity, resulting in a remarkable decrease on weight retention and molar mass. The hydrolysis degradation rate increased in the order lcP(TMC-*b*-LLA), lcP(LLA-ran-TMC), lcP(LLA-grad-TMC). Therefore, the hydrolytic degradation behavior of comb-like graft copolymers depends on both the compositions and the sequences dramatically.

## 1. Introduction

Synthetic biodegradable polymers, such as polylactide (PLA), polyglycolide (PGA), polycaprolactone (PCL) as well as their copolymers, have been proverbially studied and widely used in biomedical applications [1,2,3]. Among them, PLA is a very promising material because it combines biodegradability, biocompatibility, and excellent processability, while it is derived from natural resources [4]. Hence, PLA has been considered as an ideal biomaterial for biomedical and pharmaceutical applications, especially in tissue engineering and controlled drug delivery.

To be used in the biomedical field, polymers must generally meet strictly property requirements. Consequently, the improvement of properties appears necessary for most application fields. It has been reported that the molecular chain structure, including chemical composition, sequence structure, and topology of the copolymers, have an impact on the final properties [5,6]. A variety of molecular architectures has been proposed to enhance or modify the properties of PLA materials, such as star-shaped, grafted, hyperbranched, dendritic and even cross-linked [7,8,9,10,11]. Our group had synthesized poly(l-lactide) (PLLA) with well-defined linear-comb or star-comb structures. The reports indicated that PLLA’s crystallization, rheological and thermal behaviors depended on the structures of both main chain and side chain structures dramatically [12,13]. Usually, branched PLA copolymers show a faster degradation behavior than the corresponding linear polymers due to their higher amorphous character. Amorphous regions are preferentially degraded because they are more accessible to water molecules [14]. Feng Liu et al. compared the degradation behavior of diblock, triblock and four-armed PLA-*b*-PCL copolymers. The four-armed block copolymer showed the most rapid weight loss, while the diblock copolymer exhibited the slowest degradation, suggesting that molecular structure strongly affects the degradation [15]. However, PLA is known to be rather brittle and stiff to use for certain applications, such as soft tissue engineering fields [16]. Therefore, copolymerization can be an effective method to improve PLLA’s properties and reach desired degradation behavior.

Poly (1,3-trimethylene carbonate) (PTMC) is an amorphous elastomer with a relatively low glass transition temperature (*T*_g_ about −16 °C). Due to its biocompatibility and flexibility, PTMC is widely used in soft tissue engineering and drug delivery [17,18,19]. PTMC degrades by surface erosion without acidic products, that could allow to obtain zero-order drug release kinetics as well as protection of labile drug molecules [20]. In fact, several investigations concerning the degradation behaviors of PLA/PTMC copolymers have already been presented [21,22,23]. For copolymers, the properties depend not only on the molecular architecture, but also on the sequence structures of their polymer chains. Monomers can be distributed along a polymer chain statistically, grouped into blocks, repeated periodically into random, or changed progressively in composition to create a gradient. Chain sequences also affect the degradation process of copolymers a lot, which means copolymers even with similar comonomer composition may differ dramatically in degradation behavior [24,25]. However, the effects of comonomer compositions and chain sequences on the degradation behavior of graft copolymer wasn’t reported.

The objective of this research is to elucidate the effects of comonomer compositions and chain sequences on the degradation behaviors of graft copolymers. In our previous work, a series of block, gradient and random copolymer with linear-comb architectures were achieved using hydroxylated polybutadiene as the macroinitiator by simple ring-opening polymerization of LLA and TMC [26]. Herein we studied graft copolymers in vitro hydrolytic degradation, which were carried out in a pH 7.4 phosphate buffer taken as a model of biological fluids. Various analytical techniques were used to monitor the degradation behaviors, including variations of weight retention, water absorption, composition evolution, and surface morphology. Understanding the degradation mechanisms of graft polyesters will permit the prediction and the adjustment of their degradation rate, enabling the adaptation of the polymers to the requirements of a specific biomedical application.

## 2. Experimental Section

### 2.1. Materials

Butadiene (Yanshan Petrochem. Co., polymerization grade) was treated with little of *n*-butyllithium (*n*-BuLi, J&K Chemical, Shanghai, China, 2.5 M solution in *n*-hexane) to remove the moisture and inhibitor. Dichloromethane (CH_2_Cl_2_, Dalian, China), toluene (Tol., Aladdin) and cyclohexane were distilled from CaH_2_ under nitrogen. 1, 8-Diazabicyclo [5.4.0] undec-7-ene (DBU, Sigma-Aldrich, 98%) was dried over calcium hydride, distilled under reduced pressure and stored in a glovebox. l-lactide (l-LA, Jinan Daigangbio. Co., Jinan, China, 99%) and trimethylene carbonate (TMC, Jinan Daigangbio. Co., 99%) were triple recrystallized from ethyl acetate, respectively, then dried and stored in a glovebox. Trifluoromethanesulfonic acid (TfOH, Aladdin, 98%), formic acid (HCOOH, Aladdin, 88%), hydrogen peroxide (H_2_O_2_, 30%), 1,5,7-triazabicyclo [4,4,0] dec-5-ene (TBD, J&K Chemical, 98%), and other reagents were used as received without further purification.

### 2.2. Synthesis of Linear-Comb Copolymers with Different Sequence Structures

**Scheme 1:**
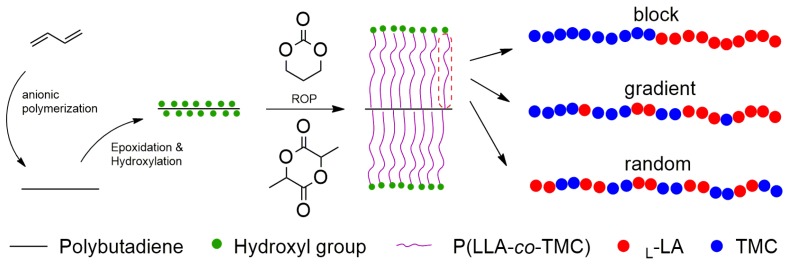
Synthesis and the structure of comb-like graft lcP(LLA-*co*-TMC): including lcP(TMC-*b*-LLA), lcP(LLA-grad-TMC) and lcP(LLA-ran-TMC) [26].

The linear-comb copolymers of TMC and LA were designed with block, gradient, and random side chains. These copolymers were all successfully synthesized as described in our previous study [26], as shown in Scheme 1.

### 2.3. Hydrolytic Degradation Procedures

Sample disks were cut into 10 mm × 10 mm × 0.4 mm size and weighted (*w*_0_). Then samples were immersed in vials containing 10 mL of 0.05 M PBS solution (pH = 7.4), which were refreshed every 72 h. The vials were placed under 37 °C. At regular time intervals, the samples were rinsed thoroughly with distilled water and weighed immediately after wiping the surface with a tissue to obtain the wet weight (*w_w_*). Next, samples were vacuum-dried at 37 °C to a constant weight (*w_d_*) before analysis. Degradation studies were performed in quadruplicate, with given data corresponding to the average values. The weight retention (%WR) and water absorption (%WA) were calculated via the following equations:

(1) $$\%\mathrm{WR}=\frac{w_{d}}{w_{0}}\times 100$$

(2) $$\%\mathrm{WA}=\frac{w_{w}-w_{d}}{w_{d}}\times 100$$

### 2.4. Measurements

The average molar mass (*M*_n_,_GPC_) and dispersity (PDI) values of the polymers were determined by gel permeation chromatography (GPC) using a Waters 1515 HPLC pump, a Waters 2414 refractive index detector, and PS columns (one PL gel 5 µm 10E4A and one Shodex KF805, Shodex, Shanghai, China) in THF as eluent at a flow rate of 0.6 mL min^−1^ at 35 °C. The calibration was based on PS standards (Shodex PS STD SM-105).

^1^H NMR and ^13^C NMR spectra were recorded on a Bruker Avance 400 MHz spectrometer at 25–30 °C in CDCl_3_ with a concentration of 4% w/v. Chemical shifts (*δ*) are reported in ppm and were referenced internally relative to tetramethylsilane (*δ* 0 ppm) using the residual ^1^H (*δ* 7.26 ppm) and ^13^C (*δ* 77.16 ppm) solvent resonances.

The thermal behavior of the polymers was measured on differential scanning calorimeter (DSC, TA, Q20). Each sample was heated to 200 °C at a heating rate of 10 °C min^−1^ in aluminum pans under a nitrogen atmosphere. Thermal history was removed by keeping the samples at 200 °C for 3 min. Then samples were cooled to −40 °C at 10 °C min^−1^, followed by heating to 200 °C at 10 °C min^−1^.

The morphological observation of the butanediol films before and after degradation was performed using a scanning electron microscope (SEM) under an acceleration of 20 kV. All the specimens were covered with a thin layer of gold before testing.

## 3. Results and Discussion

### 3.1. Characterization

Series of linear-comb poly (trimethylene carbonate)-*block*-poly(l-lactide) (lcP(TMC-*b*-LLA)) with TMC/l-LA feed ratios of 3/7, 5/5 and 7/3 were controlled synthesized. The block copolymers were named lcP (TMC-*b*-LLA)74, lcP (TMC-*b*-LLA)51 and lcP (TMC-*b*-LLA)30, by the percentage of LA. Moreover, well-defined linear-comb PLLA-gradient-PTMC and linear-comb PLLA-random-PTMC with 50% PLLA content were also controlled synthesized, named lcP(LLA-grad-TMC) 52 and lcP(LLA-ran-TMC) 50 respectively. The formation of the chain microstructures was confirmed by ^1^H and ^13^C NMR, and GPC. Linear-comb PLLA (lcPLLA) and linear-comb PTMC (lcPTMC) homopolymers were applied as comparison samples. Comonomer compositions and molecular weights of all the samples are gathered in Table 1.

### 3.2. Weight Retention and Water Absorption

**Figure 1:**
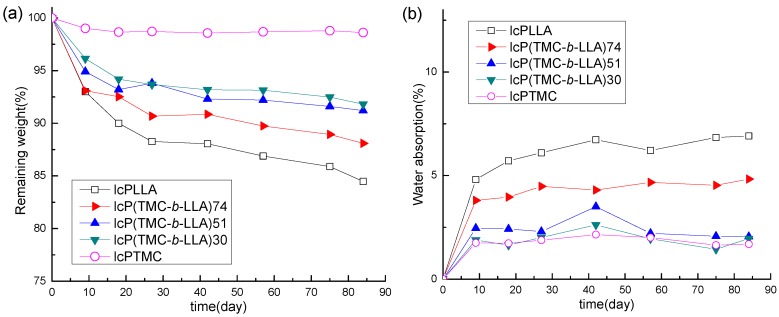
Weight retention (%WR) (**a**) and water absorption (%WA) (**b**) of the linear-comb P(TMC-*b*-LLA) copolymers.

For further biomedical applications, the studies would perform with physiological-like fluids [27]. Here, the hydrolytic degradation of the polymers was performed in pH 7.4 PBS at 37 °C. Figure 1a shows the evolution of the remaining weight of the linear-comb diblock copolymers as well as their homopolymers during 84 day-degradation. Homopolymer lcPTMC degrades extremely slow, as its remaining weight changes little during the 12 weeks. This is in agreement with the linear PTMC almost not degrade by pure hydrolysis in pH 7.4 phosphate-buffered saline (PBS) [28]. In contrast, the remaining weight of the block copolymers show some differences. Sample lcP(TMC-*b*-LLA)30 appears the most resistant to hydrolysis degradation among the block copolymers with 91.8% weight remaining after 12 weeks due to its low lactide content. Weight loss of the copolymers increases with LA content. Even though longer PLLA chain results in higher crystallinity, which would lead to slower degradation. PLLA block degraded much faster than PTMC block in the graft copolymer, no matter the PLLA parts crystallized or not. Homopolymer lcPLLA shows the fastest degradation rate with about 84.5% remaining weight at last. The higher LA content in the lcP(TMC-*b*-LLA) copolymer, the faster weight decreases, since ester bonds are more susceptible to hydrolysis than carbonate bonds [29].

Water uptake plays an important role during hydrolytic degradation, which can be caused and reflected by water absorption. Figure 1b presents the water absorption profile of the linear-comb diblock copolymers as well as their homopolymers during the degradation time. Homopolymer lcPTMC appears very hydrophobic with only 2.1% water absorption from the beginning to the end of the process. Higher water uptake is observed for diblock copolymer lcP(TMC-*b*-LLA)74 about 4.8% after 12 weeks. The other two copolymers with lower LA content show a similar low absorption level as compared to lcPTMC. Sample lcPLLA presents the highest water absorption, which under 6.9% after 12 weeks. These findings indicate that the linear-comb block polyesters are highly hydrophobic and slowly degrade during the hydrolytic degradation.

**Figure 2:**
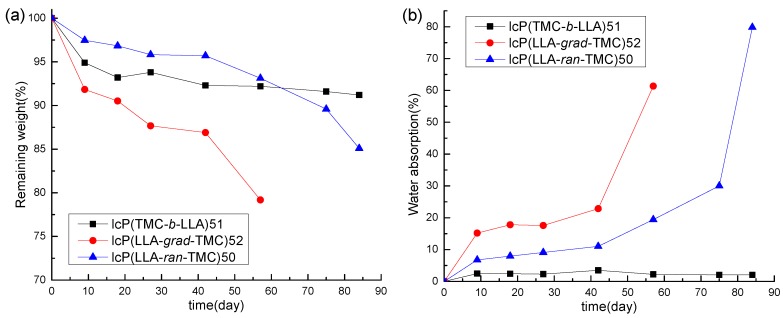
Weight retention (%WR) (**a**) and water absorption (%WA) (**b**) of the linear-comb PLLA/PTMC copolymers with block, gradient, and random sequence structures.

The linear-comb copolymers with block, gradient, and random sequences are studied to investigate the effect of side-chain sequence on the hydrolysis degradation behaviors of graft copolymers. As shown in Figure 2a,b the remaining weight and the water absorption of these graft copolymers were quite different. Unlike the linear-comb block copolymer, the random copolymer degrades constantly to reach 85.1% weight left at last. Due to the semi-crystalline morphology of the block copolymer, which will be shown below, lcP(TMC-*b*-LLA)51 degrade slower than lcP(LLA-ran-TMC)50. It is well known that water molecules can only penetrate amorphous zones of PLLA instead of compact crystalline ones. The highest weight loss was obtained by the gradient copolymer lcP(LLA-grad-TMC)52, which showed the most rapid weight loss and became too fragile to weight after 57 days. The highest weight loss and water absorption can be attributed to the gradient sequence and, more probably, to its highest PDI (2.2 versus 1.3) of the copolymers. The acceleration of water uptake observed at the later stages of degradation, in particular in the case of lcP(LLA-grad-TMC)52, can be described as releasing of soluble degradation byproducts. The soluble species leave pores or cavities in the bulk, both facilitating water absorption.

### 3.3. Molecular Weight and Composition Evolution

**Figure 3:**
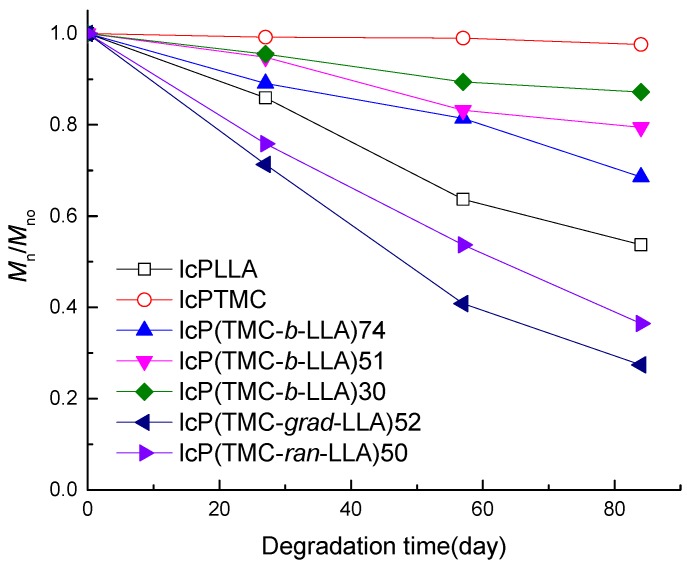
*M*_n_/*M*_n0_ decrease of linear-comb PLLA, PTMC and LA/TMC copolymers during hydrolytic degradation as a function of degradation time.

The information of number-average molar mass (*M*_n_) and the polydispersity index (PDI) during degradation were obtained via GPC monitoring. Figure 3 shows the *M*_n_/*M*_n0_ decrease during hydrolytic degradation. In order to compare the degradation rate of the graft copolymers, the exponential relationship between molecular weight and degradation time for biodegradable polyesters was used [30].

(3) $$\ln\left(\frac{M_{n}}{M_{n0}}\right)=-k\cdot t$$

The values of *k* are calculated from the slope of the fitting curve during the 84 days of study (*R*^2^ > 0.99). The lcPTMC appears non-degradable as barely any molecular weight decrease is observed after 84 days (*k* = 0.0003 days^−^^1^). In contrast, the linear-comb copolymers exhibit various degradation rates, gathered in Table 2. Obtained *k* for lcP(TMC-*b*-LLA)74, lcP(TMC-*b*-LLA) 51 and lcP(TMC-*b*-LLA)30 are 0.0040, 0.0024 and 0.0015 days^−^^1^, respectively. The higher content of TMC in the lcP(TMC-*b*-LLA) copolymer, the slower the *M*_n_ decreases, in agreement with the non-degradability of PTMC. The *M*_n_ of block copolymers decrease less than 32% of the original value after 84 days. The *M*_n_ of lcP(LLA-grad-TMC)52 and lcP(LLA-ran-TMC)50 decrease 73% and 64% of the initial molar mass, respectively. The gradient copolymer (*k* = 0.0171 days^−1^) and random copolymers (*k* = 0.0125 days^−1^) with similar composition exhibit a higher *M*_n_ decrease rate than all the block copolymers. The rapid degradation can be attributed to the decrease of chain regularity and crystallinity. This finding shows that the sequence structure of the copolymer may have a stronger effect on the degradation behaviors than the composition of the copolymer has.

Correspondingly, the PDI of all the copolymers in Table 2 shows similar increase trend during the 84 days degradation except the gradient copolymer. After water permeation of the copolymer, ester bonds and carbonate bones in the polymer chains break into hydroxyl and carboxyl end groups. These internal autocatalytic effects accelerate scission and disentanglement of polymer chains to increase PDI as previous reported [23]. Among them, the PDI of lcP(LLA-ran-TMC)50 exhibits a dramatical growth with the most rapid degradation rate. The PDI values of the sample lcP(TMC-*b*-LLA)51 and lcP (TMC-*b*-LLA)30 increases slightly. It is for the low degradation rate of block copolymers with lower LLA content. As the weight retention shows in Figure 1a, the degradation time in this study is not long enough to observe an obvious PDI change of block graft copolymers with lower LLA content. In contrast, the gradient copolymer shows a decreased trend from the beginning. The initial PDI of lcP(LLA-grad-TMC)52 is the largest (PDI = 2.2), and it decreases to 1.9 after 57 days, and to 1.8 after 84 days. The decrease of PDI could be assigned to the release of soluble species and oligomers.

**Figure 4:**
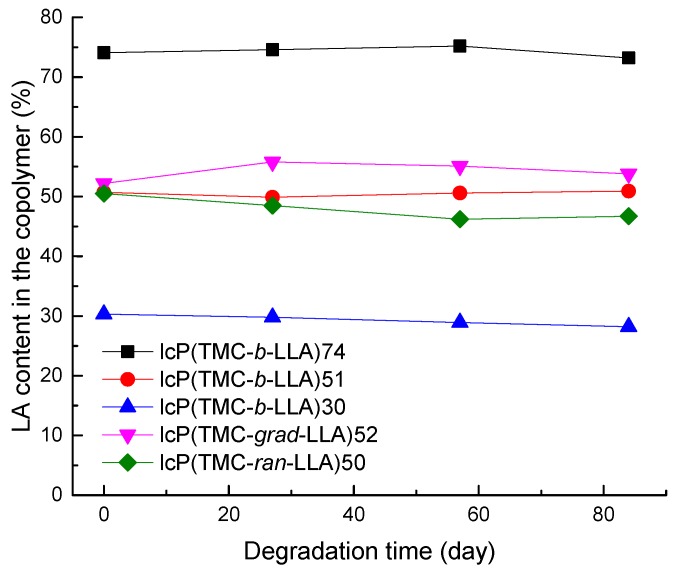
Changes of LA content in the linear-comb PLLA/PTMC copolymers during hydrolytic degradation.

The Detailed analysis of compositional changes in the copolymers during degradation has been monitored by ^1^H NMR. The LA contents of linear-comb PLLA/PTMC copolymers with different sequence structures remain constant during the degradation period, as shown in Figure 4. This finding could be assigned to the loss of TMC components together with the degradation of LA moieties, even PTMC itself does not degrade in phosphate-buffered saline without enzyme. Similar findings have also been reported in the case of other polylactide copolymers [22,31]

### 3.4. Thermal Analysis and Visual Examination

**Figure 5:**
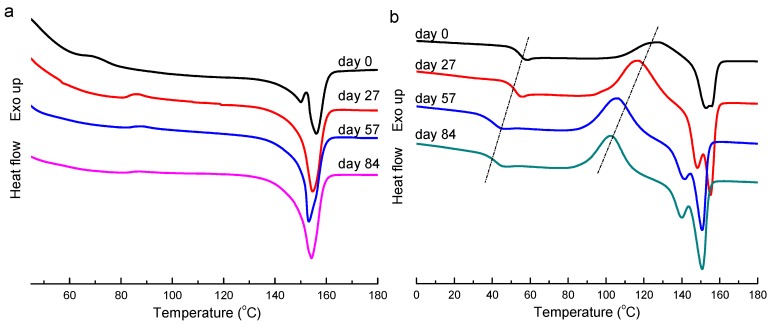
Differential scanning calorimeter (DSC) evolution of lcP(TMC-*b*-LLA)74 copolymer during degradation: (**a**) first heating, (**b**) second heating.

Figure 5 shows the DSC curves of the first and the second scans of lcP(TMC-*b*-LLA)74 at different degradation times. The thermal property changes of linear-comb PLLA/PTMC copolymers are summarized in Table 3.

During the hydrolytic degradation process, the corresponding melting temperature (*T*_m_) of lcP(TMC-*b*-LLA)74 moves obviously toward lower temperatures 154.2 °C at day 0 to 150.8 °C at day 84. The melting enthalpy (Δ*H*_m_) increases from the initial value of 29.2 to 44.8 J/g at the end of the degradation. As the degradation occurs, the polymer chains become shorter, and the chain mobility is favored to rise crystallinity. Although the Δ*H*_m_ increases, the created crystallites may become more disordered and imperfect, which leading to a lower *T*_m_. Regarding glass transition temperature (*T*_g_) behavior, lcP(TMC-*b*-LLA)74 exhibits a single *T*_g_ which decreases as the degradation time increases. The disassemble oligomers act as internal plasticizers, resulting in a decline of *T*_g_ in agreement with the PDI increase. Similar trends are obtained from lcP(TMC-*b*-LLA)51 and lcP(TMC-*b*-LLA)30 block copolymers. Their *T*_g_ and *T*_m_ decrease, in the meantime Δ*H*_m_ increase with the degradation time. The higher LA content in the block polymer, the higher initial *T*_g_, *T*_m,_ and Δ*H*_m_ the copolymers exhibit.

The thermal properties during degradation are strongly affected by LA content, and also the sequence structures. The random copolymer lcP(LLA-ran-TMC)50 doesn′t show any melting peak during the degradation. Its *T*_g_ decreases from 14.3 °C to 6.1 °C as the degradation time increases from the beginning to day 84. The disassemble oligomers act plasticizing effect, resulting in a decline of *T*_g_ in agreement with the PDI increase.

Interestingly, the gradient copolymer lcP(LLA-grad-TMC)52 doesn’t have *T*_m_ before degradation procedure. During the degradation, DSC curves show *T*_m_ of 152.3 °C on day 27 and decrease to 149.1 °C at day 84. A peak of Δ*H*_m_ also shows up on day 27 and then decreases from the initial value of 10.2 J/g to 3.4 J/g. It indicates the increased regularity of PLLA segments happened in the gradient copolymer along with degradation. This could be attributed to the rapid rupture of PTMC and amorphous region of PLLA segments, which promote additional crystalline regions formation. During this period, the decrease of PDI (Table 2) means low molecular weight oligomers are released outside. As a result, *T*_g_ increases a little bit from the initial 17.0 °C to 17.5 °C on day 57 as the plasticizing effect becomes diminished.

**Figure 6:**
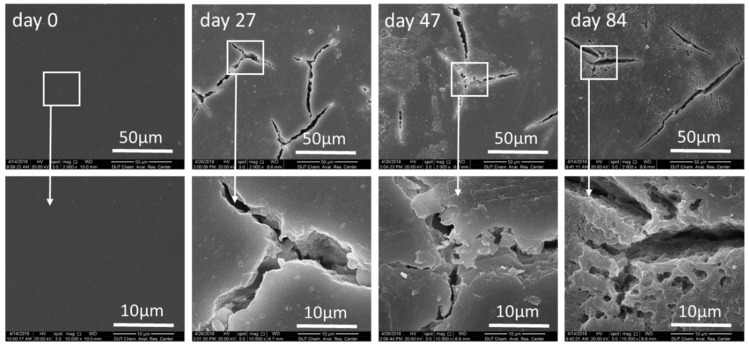
SEM images of copolymer during degradation (lcP(TMC-*b*-LLA)51).

SEM is used to monitor the film surface morphology changes during hydrolytic degradation. Figure 6 shows the SEM photographs of lcP(TMC-*b*-LLA)30 specimens after 0, 27, 47 and 84 days degradation. The copolymer presents a perfectly smooth surface before degradation. After 27 days of degradation, the surface appears slightly eroded to leave some cracks. At day 47, surface erosion continues, and the surface becomes more rugged. After 84 days, cracks grow deeper and larger, porous structures are also observed around cracks on the surface.

**Figure 7:**
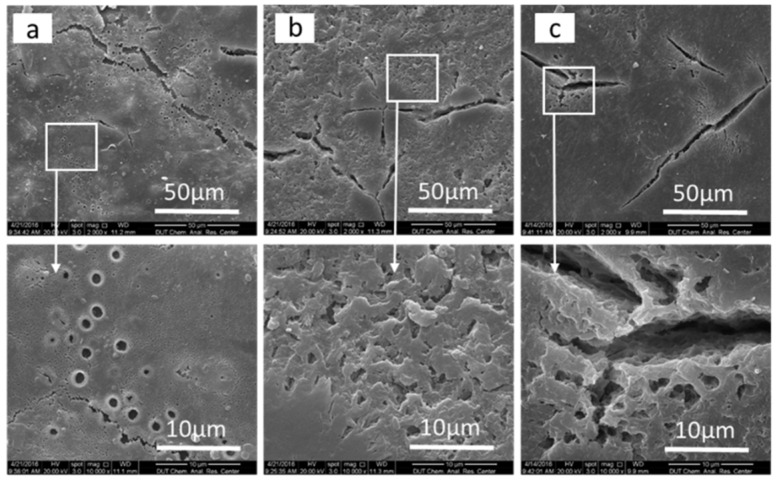
SEM images of linear-comb copolymers after 84 days degradation: (**a**) lcP(TMC-*b*-LLA)74, (**b**) lcP(TMC-*b*-LLA)51, (**c**) lcP(TMC-*b*-LLA)30.

Figure 7 shows the SEM photographs of linear-comb PLLA/PTMC block copolymers with different compositions after 84 days of degradation. For the surface of lcP(TMC-*b*-LLA)74 (Figure 7a), there are lots of cracks and tiny holes. These are the results and the reasons for water molecular entering the polymer substance. Also, the small pores on the block copolymer surface prove the low molecular substances are peel dissolving. Similarly, the surface of sample lcP(TMC-*b*-LLA)51 is rough and full of cracks (Figure 7b). In contrast, the surface of lcP(TMC-*b*-LLA)30 is rather smooth with little cracks after degradation (Figure 7c). Compared with lcP(TMC-*b*-LLA)74 and lcP(TMC-*b*-LLA)51, lcP(TMC-*b*-LLA)30 is the least degraded with the least PLLA composition in agreement with the smallest mass loss (Figure 1). Therefore, linear-comb PLLA/PTMC block copolymers are degradable in phosphate-buffered saline without enzyme, especially for those with high LA contents.

**Figure 8:**
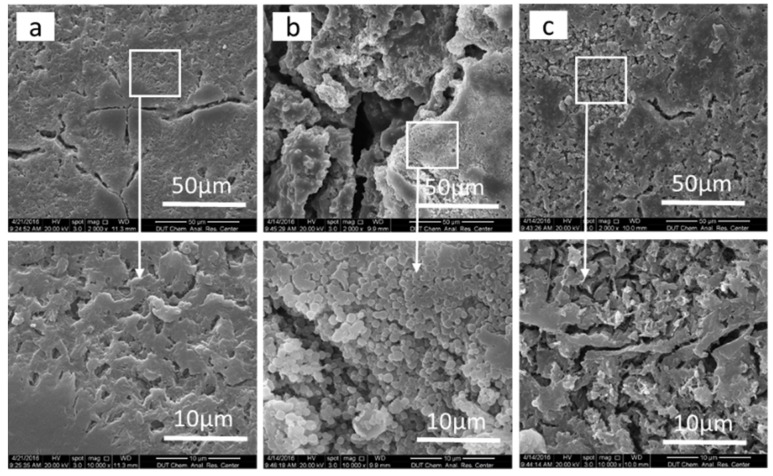
SEM images of samples after 84 days: (**a**) lcP(TMC-*b*-LLA)51, (**b**) lcP(LLA-grad-TMC)52, (**c**) lcP(LLA-ran-TMC)50.

Figure 8 shows the SEM photographs of linear-comb copolymers with block, gradient and random sequences after 84 days degradation. Unlike the linear-comb block copolymers, the linear-comb gradient copolymer is greatly degraded with large holes observed after 84 days of degradation. The surface of lcP(LLA-grad-TMC)52 appears highly porous with numerous tiny spherulites of ca. 2 μm (Figure 8b). The spherulites are PLLA crystals, which are formed during degradation in agreement with the thermal properties of lcP(LLA-grad-TMC)52 (Table 3). It was reported that initially amorphous copolymers containing larger amounts of LA units were able to crystallize during degradation because of the presence of relatively long LLA blocks [25]. The boundaries between spherulites become clearly distinguishable. Since boundaries are mainly composed of amorphous material or crystallite defects, which are already degraded and removed after 84 days. The linear-comb random copolymer lcP(LLA-ran-TMC)50 was strongly eroded to leave some pores and sponge-like structures (Figure 8c). No apparent spherulites were observed due to its random sequence structure. These findings well corroborate the DSC results of lcP(LLA-ran-TMC)50. After 84 days of degradation, the surface appeared largely eroded with some microdomains.

Comparison on the superficial morphology of these three copolymers with different sequence structures shows that the surface characteristics of lcP(LLA-grad-TMC)52 changes most obviously, with the largest holes and cracks. The film surface corrodes variously with similar comonomer composition, indicating the deformation correlates with the sequence structures.

## 4. Conclusions

Two groups of graft copolymers were analyzed during hydrolytic degradation: a series of linear-comb PLLA/PTMC block copolymer with different comonomer compositions (TMC/l-LA = 3/7, 5/5, 7/3), and three kinds of linear-comb PLLA/PTMC copolymers with similar composition (TMC/l-LA = 5/5) in block, gradient and random sequences.

In the case of linear-comb block copolymers, the degradation rate increases with the PLLA content. Since LA units are preferentially degraded during hydrolytic degradation. The weight retention and molar mass decrease due to the hydrolytic chain cleavage in the bulk. Little compositional changes are obtained during degradation, which could be assigned to the loss of TMC components together with the degradation of LA moieties. Thermal property changes are observed with decreased *T*_m_ and increased Δ*H*_m_ in all cases, which strongly supports a bulk erosion mechanism, in agreement with SEM observations. Different degradation behaviors are observed for the linear-comb block, gradient and random copolymers with similar LA content. The degradation rates of lcP(LLA-grad-TMC)52 and lcP(LLA-ran-TMC)50 are accelerated due to the loss of regularity and crystallinity, resulting in a remarkable decrease on weight retention and molar mass. The gradient copolymer lcP(LLA-grad-TMC)52 exhibits the most rapid degradation rate, which is attributable to the highest PDI. SEM observation shows the film surface corroded variously for the three kinds of linear-comb copolymers, indicating the deformation correlated with the sequence structures.

It is concluded that the degradation rate can be justified through the variation of comonomers compositions and sequence structures. Understanding the degradation mechanisms of graft copolyesters will permit the prediction and the adjustment of their degradation rate, enabling the adaptation of the polymers to the requirements of a specific biomedical application.

## Figures and Tables

**Table 1:** Characteristics of linear-comb poly(l-lactide) (PLLA)/poly (1,3-trimethylene carbonate) (PTMC) copolymers with block, gradient, and random sequences structures [26].

Sample	Feed Ratio (TMC/l-LA)	Molar Ratio (TMC/l-LA) *^a^*	*M*_n,GPC_*^b^* (kg mol^−1^)	PDI *^b^*
lcPLLA	0/100	0/100	51.2	1.4
lcPTMC	100/0	100/0	50.1	1.4
lcP (TMC-*b*-LLA)74	30/70	26/74	57.6	1.3
lcP (TMC-*b*-LLA)51	50/50	49/51	48.2	1.4
lcP (TMC-*b*-LLA)30	70/30	70/30	44.4	1.4
lcP (LLA-grad-TMC)52	50/50	48/52	38.7	2.2
lcP (LLA-ran-TMC)50	50/50	50/50	44.7	1.3

*^a^* determined by ^1^H NMR. *^b^* Number average molar mass and dispersity values determined by GPC in THF vs. polystyrene standards.

**Table 2:** Molecular characteristics of linear-comb PLLA/PTMC copolymers during degradation.

Sample/Days	lcPLLA		lcPTMC		lcP (TMC-*b*-LLA)74		lcP (TMC-*b*-LLA)51		lcP (TMC-*b*-LLA)30		lcP (LLA-*g*-TMC)52		lcP (LLA-*Ran*-TMC)50	
	*M*_n_	PDI	*M*_n_	PDI	*M*_n_	PDI	*M*_n_	PDI	*M*_n_	PDI	*M*_n_	PDI	*M*_n_	PDI
0	51.2	1.2	50.1	1.3	57.6	1.2	48.2	1.3	44.4	1.3	38.7	2.2	44.7	1.3
27	44.0	1.3	49.7	1.3	51.3	2.2	45.7	1.4	42.4	1.4	27.6	2.2	33.9	1.6
57	32.6	1.8	49.6	1.3	46.9	2.2	40.1	1.4	39.7	1.4	15.8	1.9	24.0	2.3
84	27.5	1.9	48.9	1.4	39.5	2.5	38.3	1.4	38.7	1.4	10.6	1.8	16.3	2.1
*k ^a^* (day^−1^)	0.0068		0.0003		0.0040		0.0024		0.0015		0.0171		0.0120	

*^a^ k* was calculated according to Equation (1).

**Table 3:** Thermal properties of linear-comb PLLA, PTMC, and LA/TMC copolymers during degradation.

Samples	Time (Day)	*T*_m__1_*^a^* (°C)	Δ*H*_m1_ *^a^* (J g^−1^)	*T*_g2_*^b^* (°C)	*T*_m2_*^b^* (°C)	Δ*H*_m2_ *^b^* (J g^−1^)
lcPLLA	0	157.7	39.1	52.5	164.8	34.2
	27	156.8	54.9	-	164.5	56.1
	57	155.9	54.9	-	163.1	55.4
	84	154.0	52.8	-	160.5	54.5
lcPTMC	0	-	-	−18.6	-	-
	27	-	-	−16.6	-	-
	57	-	-	−16.9	-	-
	84	-	-	−17.2	-	-
lcP(TMC-*b*-LLA)74	0	156.2	30.1	44.6	154.2	29.2
	27	154.6	44.9	44.2	155.3	36.0
	57	153.1	40.8	43.0	153.7	40.6
	84	156.3	42.7	42.3	150.8	44.8
lcP(TMC-*b*-LLA)51	0	154.1	21.9	45.3	155.7	26.2
	27	159.2	25.9	45.2	155.3	28.1
	57	159.3	28.3	44.1	154.1	29.4
	84	158.1	25.6	44.0	154.1	30.8
lcP(TMC-*b*-LLA)30	0	144.2	-	−1.6	144.7	10.9
	27	143.9	-	−7.2	143.9	12.4
	57	142.3	-	−9.1	142.3	14.2
	84	141.6	-	−9.3	141.6	15.0
lcP(LLA-grad-TMC)52	0	-	-	17.0	-	-
	27	153.8	9.2	17.3	152.3	10.2
	57	152.6	10.9	17.5	150.5	10.1
	84	151.6	19.4	10.1	149.1	3.4
lcP(LLA-ran-TMC)50	0	-	-	14.3	-	-
	27	-	-	14.1	-	-
	57	-	-	12.8	-	-
	84	-	-	6.1	-	-

*^a^ T*_m1_ and Δ*H*_m1_ were determined by DSC on the first heating. *^b^ T*_g2_, *T*_m2_ and Δ*H*_m2_ were determined by DSC on the second heating.
